# Challenges and resistance mechanisms to EGFR targeted therapies in head and neck cancers and breast cancer: Insights into RTK dependent and independent mechanisms

**DOI:** 10.18632/oncotarget.28747

**Published:** 2025-06-25

**Authors:** Shreya Shyamsunder, Zhixin Lu, Vinita Takiar, Susan E. Waltz

**Affiliations:** ^1^Department of Radiation Oncology, University of Cincinnati Medical Center, Cincinnati, OH 45267, USA; ^2^Department of Cancer Biology, University of Cincinnati College of Medicine, Cincinnati, OH 45267, USA; ^3^Research Service, Cincinnati Veterans Affairs Medical Center, Cincinnati, OH 45220, USA; ^*^These authors contributed equally to this work

**Keywords:** EGFR, RTKs, breast cancer, head and neck cancer, resistance mechanisms

## Abstract

Epidermal Growth Factor Receptor (EGFR) targeted therapies have yielded variable results in clinical trials for breast and head and neck cancers, despite EGFR overexpression in these malignancies. Primary resistance to these therapies is common, with secondary resistance often arising due to the overexpression of other receptor tyrosine kinases (RTKs) and increased downstream signaling from these RTKs. Additionally, non-RTK-driven mechanisms also contribute to anti-EGFR therapy resistance. This review highlights the role of AXL, MET, and RON families of RTKs in tumor progression and resistance to anti-EGFR therapies, focusing on breast and head and neck cancers. In breast cancer, the review discusses the intricate relationship between EGFR expression and therapeutic outcomes, emphasizing the challenges and potential strategies for enhancing EGFR-targeted treatments. It details how EGFR inhibition affects tumor progression and survival in head and neck cancer, noting that small molecule inhibitors and monoclonal antibodies, such as cetuximab, can lead to trans-activation of other RTKs. The review further explores non-RTK-driven resistance mechanisms in breast cancer, including EGFR activation through EGF-related ligands, nuclear localization of EGFR, and the overexpression of resistance-conferring proteins. In head and neck cancer, resistance is also mediated by TLR4-MyD88 signaling activation, loss of tumor suppressor genes like PTEN, activating mutations in PI3K, and involvement of STAT3. By synthesizing current insights on both RTK and non-RTK mediated resistance against anti-EGFR therapies, this review aims to guide future research and improve therapeutic strategies for these cancers.

## INTRODUCTION

Receptor tyrosine kinases (RTKs) constitute a family of highly conserved proteins that are overexpressed in multiple cancers, making them prime candidates for the development of targeted therapies. RTKs are characterized by structural features, including an extracellular domain responsible for ligand binding, a transmembrane domain, and an intracellular domain with kinase activity. In their inactivated state, RTKs may exist as monomers or in a closed conformational state, but upon ligand binding, they can dimerize, leading to the phosphorylation of tyrosine residues within the intracellular domain (i.e., EGFR family members) [1]. However, some RTKs dimerize even in the absence of ligands (i.e., the insulin receptor) [2, 3] with RTK dimers formed in the absence of ligands becoming further stabilized with ligand binding [3]. Dimerization is then followed by phosphorylation events which subsequently activate downstream signaling pathways, such as Phosphatidylinositol 3-kinase/protein kinase B/mechanistic target of rapamycin (PI3K/Akt/mTOR), Janus kinase 2/signal transducer and activator of transcription (JAK2/STAT), Focal adhesion kinase (FAK), Nuclear factor-kB (NF-kB) and Mitogen-activated protein kinase/extracellular-signal-regulated kinase (RAS/MAPK/ERK), which play crucial roles in cell growth, survival, and proliferation. In cancers, RTKs often exhibit overexpression, gain-of-function mutations, chromosomal rearrangements, and genomic amplifications, resulting in their constitutive activation [4]. Consequently, the aberrant activation of signaling pathways downstream of RTKs can lead to uncontrolled cell growth contributing to tumor formation and progression.

The epidermal growth factor receptor EGFR, known as HER(Human Epidermal Growth Factor)1 is overexpressed in both breast and head and neck cancers [5, 6]. This receptor plays a crucial role in cell proliferation, angiogenesis, apoptosis inhibition, motility, metastasis, and adhesion, making it an attractive target for therapeutic intervention. Anti-EGFR therapy has shown promise in specific clinical trials for head and neck cancer. However, in breast cancer, the response to anti-EGFR therapy has been mixed owing to the diverse phenotypes and inter-tumoral heterogeneity. The occurrence of primary and secondary resistance (resistance to therapy developed after the patient’s initial response to the therapy) to EGFR targeted therapies are the emerging challenges that are faced by the patients. Treatment resistance hinders treatment efficacy and limits long-term benefits. In both breast and head and neck cancers, in addition to resistance to anti-EGFR therapy, additional obstacles include a lack of reliable biomarkers for predicting therapeutic responsiveness. Overcoming these roadblocks is essential to unleash the full potential of EGFR-directed therapy.

In this report, we aim to review RTK signaling pathways using breast and head and neck cancer as model systems, given their relatively high levels of EGFR expression. The primary focus will be on the mechanisms behind anti-EGFR resistance, which can arise from both RTKs and non-RTKs in both cancer types. By gaining a thorough understanding of these resistance mechanisms, we can gain valuable insights to inform the design of future clinical trials and facilitate the development of more targeted and effective anti-cancer therapies.

## THE ROLES OF RECEPTOR TYROSINE KINASES IN TUMOR PROGRESSION AND THERAPY RESPONSE

### Breast cancer

**Figure 1:**
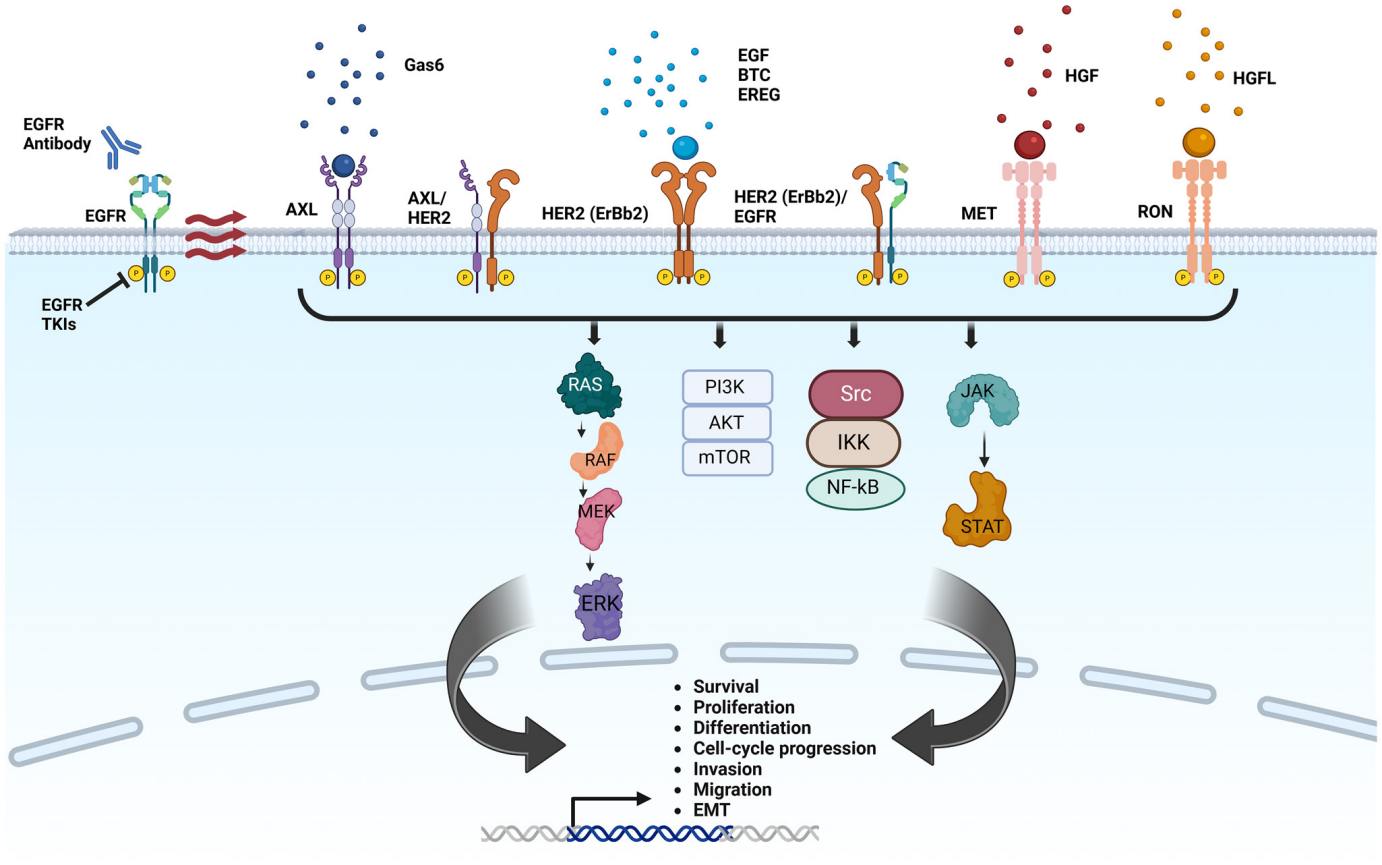
Mechanisms of anti-EGFR therapy resistance mediated by receptor tyrosine kinases in breast cancer. Treatment with EGFR TKIs or anti-EGFR antibodies leads to homodimerization or heterodimerization of EGFR family receptors (HER2) and other cell surface receptor tyrosine kinases, such as AXL, MET and RON in a ligand-dependent and ligand-independent manner. The activation of receptor tyrosine kinases will activate downstream signaling pathways, such as RAS/MEK/ERK, PI3K/Akt, NF-kB and JAK/STAT, which promote cancer survival, cell-cycle progression, invasion, and migration. Created with https://www.biorender.com/.

Breast cancer exhibits overexpression of multiple RTKs, including EGFRs, vascular endothelial growth factor receptors (VEGFRs), platelet-derived growth factor receptors (PDGFRs), insulin-like growth factor receptors (IGFRs), and fibroblast growth factor receptors (FGFRs). These receptors initiate signaling cascades that regulate cancer stemness, angiogenesis, and metastasis [3–5]. A meta-analysis involving more than 11,000 breast cancer patients revealed that elevated levels of RTKs, including HER2, EGFR and FGFR, are associated with increased breast cancer aggressiveness and decreased overall and disease-free survival [7]. The known mechanisms by which RTKs promote breast cancer progression, maintain cancer stem cell phenotypes and drug resistance involve the regulation of MAPK, JAK/STAT, and phosphoinositide 3-kinase (PI3K)/Akt pathways [8–11], as shown in Figure 1.

The unique and aberrant overexpression and/or activation profiles of RTKs in breast cancers, particularly HER2, hepatocyte growth factor receptor (MET), and EGFR, make them promising prognostic markers and therapeutic targets for disease management. The HER2 receptor, overexpressed in more than 10% of breast cancers, leading to aggressive tumor phenotypes, has been effectively targeted with Trastuzumab (Herceptin), a humanized monoclonal antibody [12, 13]. Clinical studies have shown that adjuvant chemotherapy with Trastuzumab increased patient survival and reduced the risk of death in metastatic HER2 overexpressing breast cancer [14].

MET is another receptor tyrosine kinase that plays a role in breast cancer cell growth, invasion and angiogenesis. It is recognized as a poor prognostic factor in invasive breast cancer, irrespective of other prognostic markers like HER2 and EGFR [15, 16]. These data suggest that therapies targeting MET signaling pathways may also be worth exploring as potential treatment options for MET-positive breast cancers.

The HGF (hepatocyte growth factor)/MET pathway plays an important role in normal mammary development and various breast cancer progression processes, including migration, invasion, and tubulogenesis [17–19]. Both paracrine and autocrine HGF-dependent MET signaling play significant roles in breast cancer progression [20, 21]. Specific MET mutations are also known to control the progression of primary cancers to metastatic disease; a germline mutation of MET, MET-T1010I, was found in patients with metastatic breast cancer and is sufficient to induce tumor formation and invasion *in-vivo* [22]. MET has been found to co-express and/or crosstalk with members of the EGFR family, such as HER2, in breast cancer, leading to oncogenesis and drug resistance via activation of PI3K/Akt and Ras/MAPK pathways [23]. HER2 and MET were found to be co-expressed in HER2-positive breast tumors and further abolishing MET signaling was able to sensitize these cancer cells to Trastuzumab treatment [24].

MET also co-signals with other RTKs, such as RON, also widely known as the macrophage stimulating 1 receptor (MST1R), which is overexpressed or constitutively active in more than 50% of human breast cancer cases [25]. Ligand-induced activation of MET can promote transphosphorylation of RON, which plays an important role in the MET signaling cascade [26]. RON signaling has been shown to promote mammary tumor growth and therapy resistance in various breast cancer mouse models [27]. Due to the high homology between RON and MET, they share overlapping downstream signaling pathways and have been reported to crosstalk with the same receptor tyrosine kinases [28]. The association of RON with MET and EGFR has been investigated by immunoprecipitation and cross-linking experiments, demonstrating receptor transphosphorylation, which may play a role in predicting therapeutic response and drug resistance in breast cancer [26, 29, 30].

### Head and neck cancer

**Figure 2:**
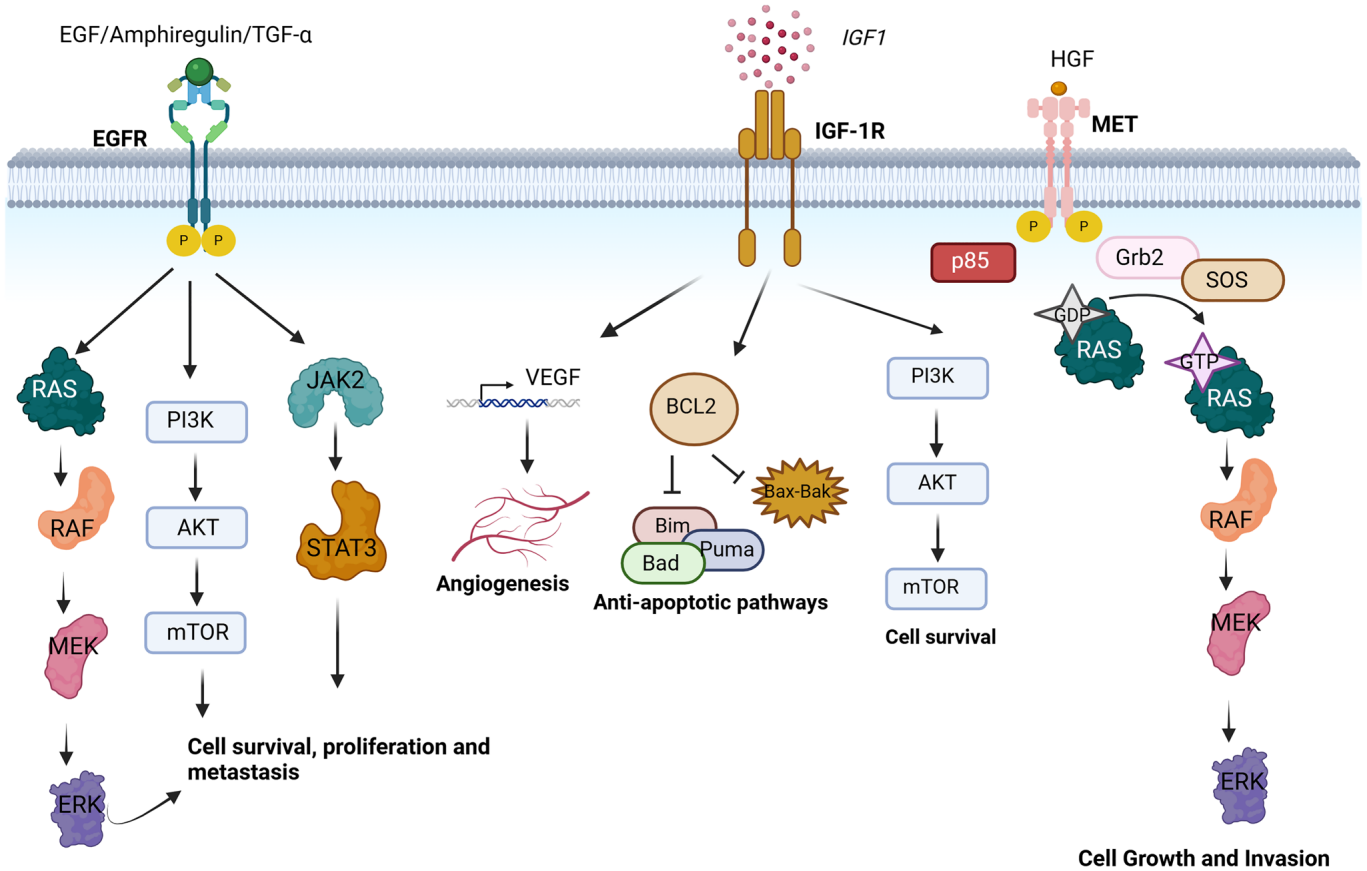
Receptor tyrosine kinase pathways mediating cancer cell survival, proliferation, invasion and apoptosis contributing to treatment resistance in head and neck cancer. EGFR is bound by ligands like EGF or Amphiregulin or TGF-α which activate downstream signaling pathways like RAS-MEK-ERK, PI3K/AKT/mTOR and JAK/STAT3 leading to cell survival proliferation and metastasis. IGF-1R when activated by IGF1 can activate anti-apoptotic pathways, increase angiogenesis and increase cell survival. c-MET activation by HGF is another mechanism by which cells continue to grow and gain invasive properties via the RAS-MEK-ERK signaling. Created with https://www.biorender.com/.

Head and neck cancer is often treated with the combination of ionizing radiation therapy and systemic therapies, including chemotherapy and/or RTK inhibitors. In head and neck squamous cell carcinoma (HNSCC), the activation of various RTKs plays a significant role in disease progression and therapeutic resistance. EGFR has been found to be overexpressed and oftentimes aberrantly activated in HNSCCs, resulting in increased proliferation and pro-tumorigenic effects [6]. Besides EGFR overexpression, other factors such as increased ligand induced activation of EGFR can also cause enhanced proliferation. Moreover, inhibiting the translation of EGFR ligands like TGF-α (produced as a result of radiation exposure) results in decreased cancer cell proliferation [6]. Preclinical studies have shown that EGFR plays a radioprotective role in head and neck cancer, leading to decreased effectiveness in response to ionizing radiation [31]. Vandetanib, a multi-RTK inhibiting drug (targeting VEGFR-2, EGFR and RET), has been shown to reverse radioresistance both *in vitro* and *in vivo* in HNSCC, when given in combination with cisplatin [32]. Vandetanib was also well tolerated when administered with cisplatin and radiotherapy, with 86.7% of patients achieving loco-regional tumor control [33]. In addition to the EGFR RTK family, other RTKs like IGF-1R are frequently activated in up to 94% of patient samples and have been implicated in driving head and neck cancer progression [34]. Apart from its role in regulating VEGF production and tumor angiogenesis, the IGF signaling axis is responsible for the activation of anti-apoptotic signaling pathways, which in turn leads to upregulation of pro-survival pathways, namely MAPK and PI3K-Akt (Figure 2). Treatment of HNSCC mouse xenografts with monoclonal antibodies targeting both IGF-1R and EGFR has provided a significant reduction in tumor volume [34, 35]. However, the mechanism by which IGF-1R crosstalks with EGFR and its impact on head and neck cancer disease progression remains elusive.

Another important RTK in head and neck cancer is MET. An upregulation of MET/HGF signaling is frequently observed. Binding of MET with its ligand HGF leads to autophosphorylation of the receptor and recruitment of signaling molecules, such as the p85 subunit of PI3K and Grb2/Sos complex which subsequently activate JNK and MAPK pathways leading to increased cell growth (Figure 2). In about 80% of head and neck cancer patient samples, MET is found to be overexpressed [36, 37]. Somatic mutations, such as Y1230C and Y1235D mutations have been detected in head and neck cancer driving invasion and an epithelial-to-mesenchymal transition (EMT) phenotype, respectively [35]. Moreover, head and neck cancer cells containing MET are more radioresistant due to the altered signaling mechanisms post irradiation [38]. MET/HGF signaling has also been shown to be responsible for the metabolic reprogramming seen in head and neck tumor cells [39]. When treated with HGF, HNSCC cells show an upregulation of glycolytic genes namely HK2, MCT1, and PFK1, indicating the glycolytic metabolism shift [40]. Additionally, the tumor microenvironment of 50% of HNSCC patients demonstrates an overexpression of HGF which has been reported to increase resistance to radiotherapy and to MET targeting tyrosine kinase inhibitor JNJ-38877605 via the association of PI3K-GAB1 [6, 41].

## APPROACHES TO TARGET EPIDERMAL GROWTH FACTOR RECEPTOR AS TREATMENT OPTIONS

The Epidermal Growth Factor Receptor family plays a crucial role in signaling pathways that regulate cell growth, survival, differentiation, and proliferation. The EGFR (ErbB) family of transmembrane receptors consists of four members, including EGFR (ErbB1), HER2 (ErbB2), HER3 (ErbB3), and HER4 (ErbB4). They play essential roles in organ development and growth [42]. However, specific members, like HER2 and EGFR, are frequently found aberrantly expressed and dysregulated in breast cancer and head and neck cancer. Under normal physiological conditions, EGFR activation occurs through dimerization with itself or other receptors in the HER family or by binding of its canonical ligands, EGF, TGF-α, or amphiregulin (AREG) (Figure 2), leading to signaling activation through MAPK, JAK/STAT or PI3K/Akt/mTOR pathways [6, 42–44].

### EGFR overexpression: A prognostic marker and therapeutic target in breast cancer

In breast carcinomas, EGFR overexpression has been associated with poor prognosis [44]. However, a number of clinical trials of EGFR-targeted therapy in breast cancer patients conducted in the 1990s and early 2000s have reported mixed results (Table 1) [50–56], partly due to the heterogeneity of breast cancer, diverse patient populations, and divergent experimental methodologies, making it very challenging to evaluate EGFR as a prognostic or predictive marker for response to targeted therapies.

**Table 1:** Clinical studies of EGFR-targeted therapies in breast cancer

Treatment	Drug class	Trial number	Trial phase	Patient selection	Therapy type	Primary endpoint	Outcome
Cetuximab	mAb	NCT00232505 [45]	II	Stage IV Triple Negative Breast Cancer (TNBC)	Mono	Overall Response Rate (ORR)	ORR: 6%
					Mono+Carboplatin		ORR: 17%
Cetuximab	mAb	NCT00463788 [46]	II	Metastatic TNBC	Cisplatin	Overall Response Rate	ORR: 10%
					Cisplatin+Cetuximab		ORR: 20%
Cetuximab	mAb	N0436 [47]	II	Metastatic BC	Cetuximab+irinotecan	Overall Response Rate	ORR: 11%
Panitumumab	mAb	NCT00894504 [48]	II	Metastatic TNBC	Panitumumab+ gemcitabine/carboplatin	Overall Response Rate	ORR: 42%
Cetuximab	mAb	NCT00600249 [49]	II	TNBC	Cetuximab+docetaxel	Pathological Complete Response (pCR)	pCR: 24%
Gefitinib	EGFR TKI	NCT00066378 [50]	II	HR+Metastatic BC	Anastrozole+placebo	Progression-Free Survival (PFS)	PFS: 32%
					Anastrozole+Gefitinib		PFS: 32%
Gefitinib	EGFR TKI	NCT00229697 [51]	II	ER+Metastatic BC	Tamoxifen+placebo	Progression-Free Survival (PFS)	PFS: 8.8%
					Tamoxifen+Gefitinib		PFS: 10.9%
Gefitinib	EGFR TKI	NCT00239343 [52]	II	Stage II–IIIA BC	Epirubicin+Paclitaxel+ Gefitinib	NA	NA
					Epirubicin+Paclitaxel+ Placebo		
Gefitinib	EGFR TKI	NCT00057941 [53]	II	Stage I to IIIB hormone receptor-positive BC	Anastrozole+placebo	Overall Response Rate	ORR: 61%
					Anastrozole+Gefitinib		ORR: 48%
Erlotinib	EGFR TKI	NCT00739063 [54]	II	Metastatic BC	Monotherapy	Overall Response Rate	ORR: 3%
Afatinib	EGFR TKI	NA [55]	II	HER2-, ER-, and/or PgR-negative BC	Monotherapy	Overall Response Rate	ORR: 0%
Afatinib	EGFR TKI	NCT01125566 [56]	III	HER2-overexpressing metastatic BC	Afatinib+vinorelbine	Median PFS months	Median PFS months: 5.5
					Trastuzumab+vinorelbine		Median PFS months: 5.6

In the late 1980s, Sainsbury and colleagues evaluated 135 primary breast cancer samples, 76 of which had available nodal and EGFR status [5]. They found that EGFR-positive tumors were associated with worse relapse-free survival (RFS) and overall survival (OS) compared to EGFR-negative tumors. EGFR expression was inversely correlated with hormone receptor (estrogen receptor and progestogen receptor) status, suggesting that incorporating EGFR status as a prognostic factor could provide more precise prediction of therapy response and RFS/OS in both hormone-receptor-positive and hormone-receptor-negative breast cancer patients.

Toi and colleagues subsequently linked EGFR expression to cell proliferation of breast tumors and found enhanced EGFR expression at metastatic tumor sites compared to primary sites, suggesting its role in promoting tumor metastasis [57, 58]. They also demonstrated that EGFR status, in combination with HER2 status, can act as an effective predictor of a high risk of relapse. However, not all breast cancer patients with EGFR-expressing tumors respond to EGFR-targeted therapy, indicating that simply inhibiting EGFR may not be sufficient to achieve therapeutic benefit in breast cancers [59]. Despite the variability in response among breast cancer patients with EGFR-expressing tumors to EGFR-targeted therapy, a study by Lee’s group revealed that pre-treatment with EGFR-targeted therapy in specific subsets of TNBC can sensitize tumor cells to cytotoxic therapy by transcriptionally rewiring cells to a pro-apoptotic state [60]. TNBC is defined by the absence or minimal expression of estrogen receptor (ER), progesterone receptor (PR), and human epidermal growth factor receptor 2 (HER2) [61]. TNBC is a very aggressive BC subtype, often leading to higher rates of recurrence and poorer overall survival compared to other breast cancer subtypes This finding suggests the importance of targeting the proper patient population to achieve the therapeutic benefit of EGFR-targeted therapy in breast cancer patients [62, 63].

EGFR has been identified as an additional cancer-specific target to reduce systemic toxicity and increase tumor specificity for chemotherapy drugs [64–66]. Clinical data has proven the feasibility of EGFR inhibition in combination with taxane or cisplatin in TNBC. This combination therapy provides patients with a longer progression-free survival compared to cisplatin alone [46, 67]. Researchers developed strategies to improve the efficacy of doxorubicin (DOX) in breast cancer treatment. These include conjugating DOX to EGFR-binding peptide (EBP) and creating anti-EGFR immunoliposome-doxorubicin. The latter covalently links Fab′ fragments of anti-EGFR antibody (cetuximab) to liposomes containing probes and doxorubicin [68–70]. In the breast cancer xenograft models used in these studies, treatment with either DOX−EBP conjugate or anti-EGFR immunoliposome-doxorubicin resulted in tumor regression and was significantly superior to treatment with non-targeted groups. A phase II trial conducted last year, testing anti-EGFR-immunoliposomes loaded with doxorubicin in patients with advanced TNBC, did not reach its primary endpoint due to low efficacy [71]. Nevertheless, exploring different formulations and drug delivery methods remains promising for other EGFR-expressing cancers, such as head and neck cancer, where EGFR targeting has already demonstrated anticancer effects.

MET has been identified as a key regulator of EGFR tyrosine kinase inhibitor resistance in various cancer types [72–74]. A physical and functional interaction between EGFR and MET was discovered in breast cancer cells [75]. This interaction, mediated by Src activity, regulates the phosphorylation of both EGFR and MET independently of EGFR kinase activity [76]. The association between EGFR, MET, and Src, which regulates growth in breast cancer cells, underscores the need to identify active receptor co-activation processes.

Lipid rafts have been proposed as a model and platform for the interaction of EGFR and Src, leading to the activation of EGFR-kinase-independent survival signaling in breast cancer cells [77–79]. EGFR localization to lipid rafts was first found correlated with EGFR TKI resistance in TNBC cell lines, promoting EGFR-dependent activation of the Akt pathway [78]. Further research demonstrated that the non-receptor tyrosine kinase Src co-localizes and co-associates with EGFR within the lipid rafts. The mechanisms by which lipid rafts regulate EGFR signaling to Akt was Src kinase-dependent in the Sum159 cell line [79].

To further explore the therapeutic value of targeting EGFR in breast cancer and its potential role as a chemosensitizer, several steps are important. These include optimizing the selection of breast cancer patients, improving the specificity of drug conjugates, considering rational combinations of therapy (i.e. NCT04485013) and designing sequential treatment regimens in clinical trials. Additionally, identifying other molecular markers will be crucial. Studying the potential interaction of EGFR with other RTKs and non-RTKs, such as MET, IGF-1R and Src, which have been linked to resistance to EGFR targeted therapies, will be a viable approach in enhancing treatment outcomes.

### The effects of EGFR inhibition on tumor progression and survival outcomes in head and neck cancer

In over 80–90% of head and neck squamous cell carcinomas (HNSCC), there is an overexpression of EGFR and its ligands [43, 80]. The increase in expression of EGFR ligands such as TGF-α and amphiregulin among others can be a result of human activities like tobacco smoking [43]. EGFR is a transmembrane receptor which has the ability to translocate to the nucleus upon interaction with anti-EGFR antibodies like cetuximab, EGFR ligands, Src kinases and Epstein Barr Virus [43]. Once in the nucleus, EGFR can either bind to and activate multiple cell cycle regulating genes downstream, or cause phosphorylation of proteins like DNA-PK owing to its activity as a tyrosine kinase. These activities can ultimately result in repair of damaged DNA in the cancer cells and thereby increase cellular proliferation rate resulting in acquired resistance to treatment [43]. Thus, EGFR inhibition has been evaluated as a potential treatment option in head and neck cancers.

There are two main types of EGFR targeted inhibitors: (1) small molecule EGFR-targeted tyrosine kinase inhibitors and (2) EGFR targeted monoclonal antibodies. The tyrosine kinase inhibitors compete with ATP at the tyrosine kinase domain in the intracellular region of EGFR and inhibit downstream signaling pathways. Although they tend to have short half-lives, they offer the advantage of oral administration and fewer inflammatory hypersensitivity reactions. Lapatinib has been shown to be effective in inhibiting proliferation of Human Papillomavirus (HPV) positive head and neck cancer by decreasing the expression of E6 and E7 oncogenes and inhibiting phosphorylation of Akt, leading to subsequent inactivation of HER2 and EGFR signaling pathways that play a role in cell proliferation [81]. However, the efficacy of lapatinib as a monotherapy has been dismal. The results of a phase II trial in recurrent/metastatic (R/M) head and neck cancer showed that as a monotherapy, lapatinib was not beneficial to patients, even those with previous exposure to anti-EGFR therapies [82]. A randomized study conducted by Harrington et al. showed that a combination of lapatinib with cisplatin based chemoradiotherapy was better tolerated and increased complete response rate and progression free survival even in HPV-negative head and neck cancer [83]. Erlotinib, another EGFR inhibitor, has been shown to induce cell cycle arrest in HPV negative HNSCC cells in G2 phase and has shown inhibitory effects on double strand break repair proteins like PARP1, leading to increased radiosensitivity [84]. In *in vitro* and *in vivo* studies, the pan-EGFR TKI afatinib has shown better therapeutic effect on HNSCC when compared to erlotinib. Afatinib treatment also has been found to inhibit the DNA damage repair proteins and radio-sensitize HNSCCs by inhibiting Oct3⁄4 and CD44 markers on radiation induced cancer stem cells [85].

The second approach to inhibiting EGFR is with monoclonal antibodies which bind to and block the receptor-ligand interaction, leading to inhibition of EGFR dimerization. The receptor antibody complex eventually gets internalized and degraded, causing the downregulation of EGFR. Cetuximab is a therapeutic monoclonal antibody which binds to EGFR with an affinity of five to ten times that of canonical EGFR ligands [86]. Cetuximab has shown inhibitory effect of HNSCC cell proliferation *in vitro* and has evidence of promoting antibody dependent cell-mediated cytotoxicity via cytotoxic immune cells. In a randomized phase III clinical trial, Vermorken et al., demonstrated that a combination of cetuximab and platinum-based chemotherapy improved overall survival in recurrent/metastatic HNSCC compared to standard of care combination of platinum-based therapy with fluorouracil [87]. In the curative setting, Bonner et al. showed that cetuximab given in combination with radiation reduced the risk of cancer related death by 26%, improved the median overall survival (49 months) compared to radiation alone (29.3 months) and the combination also prolonged progression-free survival [88]. Several other EGFR targeting monoclonal antibodies like zalutumumab and panitumumab have been evaluated in HNSCC. However, panitumumab was not as effective as cetuximab, possibly due to its inability to mimic cetuximab’s effect of promoting the antibody dependent cellular cytotoxicity (ADCC) and activation of NK cells [89]. Thus, for head and neck cancer, cetuximab is the only therapeutic monoclonal EGFR targeting antibody approved by the FDA [43]. Clinical trials combining cetuximab with other chemotherapies, ionizing radiation and certain targeted therapies have led to better survival outcomes for patients with recurrent/metastatic (R/M) HNSCC (Table 2) [90–95].

**Table 2:** Clinical studies combining cetuximab with other chemotherapies and ionizing radiation in head and neck cancer

Treatment	Trial number	Trial phase	Patient selection	Therapy type	Primary endpoint	Outcome
Cetuximab+ Erlotinib+ chemotherapy (carboplatin, paclitaxel)	NCT01316757 [91]	II	Recurrent/metastatic (R/M) HNSCC	Cetuximab+carboplatin+ paclitaxel	Objective Response Rate (ORR)	ORR of 33.3%
				Erlotinib+Cetuximab+ carboplatin+paclitaaxel		ORR of 62.5%
Cetuximab+ chemotherapy (Carboplatin/Cisplatin and 5-Fluorouracil)	NCT00122460 [87]	III	Recurrent/metastatic (R/M) HNSCC (EXTREME)	Cetuximab+ Chemotherapy	Overall survival	Median Overall survival: 10.1 months
				Chemotherapy alone		Median Overall survival: 7.4 months
Paclitaxel+ Carboplatin+ Cetuximab	UMIN000010507 [90]	II	Recurrent/metastatic (R/M) HNSCC (EXTREME)	Paclitaxel+Carboplatin+ Cetuximab	Overall response rate	ORR of 40%
Cetuximab+ Cisplatin+ Radiation	NCT00096174 [92]	II	Locally Advanced Head and Neck Squamous Cell Carcinoma	Cetuximab+Cisplatin+ Radiation	2-year progression-free survival (PFS)	Median PFS: 19.4 months, 2-year PFS: 47%
IMC-A12 (Cixutumab)+ Cetuximab	NCT00617734 [93]	II	Recurrent/metastatic (R/M) HNSCC	IMC-A12	Progression-Free survival	1.9 months
					IMC-A12+Cetuximab	2.0 months
Cetuximab+ Bevacizumab	NCT00409565 [94]	II	Recurrent/metastatic (R/M) HNSCC	Cetuximab+Bevacizumab	Objective response rate	ORR of 16%
Cetuximab+ Sorafenib	NCT00815295 [95]	I B/II	Recurrent/metastatic (R/M) HNSCC	Cetuximab+Sorafenib	Tumor control rate	43.8%

Compared to healthy epithelial tissue, head and neck squamous epithelium tends to increase complement activation (which canonically is a component of innate immunity that is activated upon microbial infection or in autoimmune diseases). Cell lines sensitive to EGFR inhibition show a high level of complement activation following EGFR inhibitory treatment. Upon testing for complement activation in cetuximab-resistant cell lines with human serum, there was an increase in complement component C3 and Terminal Complement Complex [96]. This complement activation as a consequence of anti-EGFR therapy, can exacerbate cutaneous toxicity and can potentially be a cause of increased inflammation in the tumor microenvironment, contributing to treatment resistance.

The activation of the EGFR and PI3K pathways differs based on the HPV status of the head and neck cancer. HPV-positive HNSCC tumor samples show significantly reduced EGFR and PI3K pathway activation. On the contrary, it was found that 56% of HPV-negative HNSCC tumor samples showed activation of EGFR or PI3K pathways [97]. The high expression of EGFR is also associated with keratinization status and is indicative of poorer prognosis irrespective of the tumor’s HPV status. Head and neck tumors which express high EGFR, also show concomitant expression of pAKT which overall has a detrimental influence on patient survival [98]. Thus, it is easy to appreciate the multi-faceted role played by EGFR in head and neck cancer progression and treatment resistance.

## MECHANISMS OF ANTI-EGFR RESISTANCE MEDIATED BY RTKS IN BREAST CANCER

Resistance to EGFR–tyrosine kinase inhibitor (EGFR-TKIs) therapies in breast cancer presents a significant clinical challenge, with the mechanisms of this resistance remaining largely unknown.

Clinical trials with the EGFR TKI gefitinib in breast cancer have shown limited clinical responses and a low disease control rate, particularly in triple-negative breast cancer, despite the higher degree of EGFR overexpression in this subtype [64, 99, 100]. One explanation for the limited success of EGFR TKIs in breast cancer is that following EGFR inhibition, other receptors and/or cell surface proteins activate and/or heterodimerize, which provides alternative growth and survival signaling cascades downstream of EGFR, such as the MEK/MAPK pathway [101] (Figure 1). Therefore, understanding the molecular mechanisms beyond EGFR is crucial, especially in cases where EGFR signaling is absent. Crosstalk between EGFR with other receptor tyrosine kinase can lead to the activation of survival signaling pathways and contribute to the resistance phenotype. Studying the interactions and cross-talks may provide insights into the development of anti-EGFR therapies.

### Activation of insulin growth factor receptor and MET/RON signaling

In human breast and prostate cancer cells, elevated levels of activated IGF-1R, Akt, and protein kinase C (PKC) delta have been associated with acquired resistance to EGFR TKIs [102]. The activation of IGF-1R and downstream signaling molecules is believed to promote survival pathways, leading to resistance. Additionally, in EGFR TKI-resistant breast cancer cell lines, the receptor tyrosine kinase MET plays a role in promoting resistance. The activation of Src and MET/Src signaling mediates EGFR tyrosine phosphorylation and promotes cell growth even in the presence of EGFR TKIs [75]. Such aberrant EGFR phosphorylation, caused by direct crosstalk between EGFR and other receptor tyrosine kinases or indirectly with the help of Src creating docking sites for EGFR interaction, is considered a mechanism leading to intrinsic resistance of breast cancer to EGFR TKIs. The aberrant signal transduction in cancers mediated through RTK crosstalk contributes to tumorigenesis and therapy-resistance, which poses challenges for the development of effective therapies.

**Figure 3:**
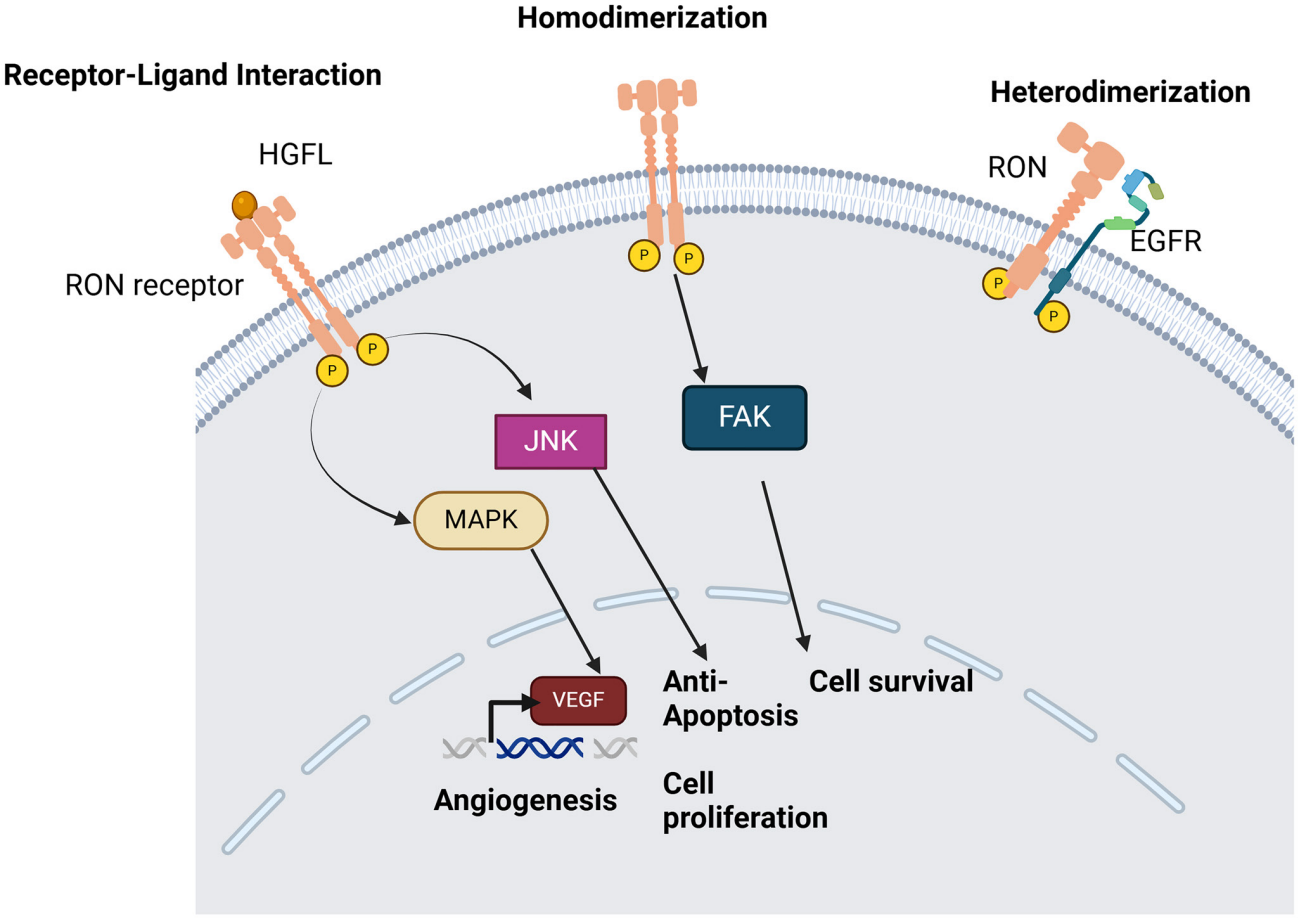
Mechanisms of RON activation which aid in cell survival and resistance to apoptosis. The RON receptor tyrosine kinase can get activated by extracellular ligands, homodimerization and heterodimerization with EGFR, leading to increased angiogenesis, resistance to apoptosis and cell survival. Created with https://www.biorender.com/.

Given the multiple signaling pathways likely contributing to therapeutic resistance, identifying the molecular mechanisms by which cells survive despite EGFR inhibition and then employing a multiple-pronged strategy to target these pathways will be required for improved therapy outcomes. For example, preclinical studies using a TNBC PDX model have shown that combined inhibition of MET and EGFR is more effective than monotherapy [103]. This finding indicates that targeting both MET and EGFR signaling might offer a potential strategy to overcome resistance, in tumors that have ‘secondary’ cell-survival-sustaining RTKs. MST1R/RON emerged as a potential therapeutic target in various cancers because its activation has been implicated in contributing to resistance to treatment failure and resistance to cell death after chemotherapy or targeted therapies [8]. RON can be activated via extracellular ligands, homodimerization, and heterodimerization with EGFR, further leading to cell survival and resistance to apoptosis (Figure 3).

### AXL-mediated downstream signaling

In HER2+ breast cancer, AXL forms a complex with HER2 (Figure 1), promoting its stability and recruitment at the cell surface, thereby promoting the metastatic cascade in cancer progression [104]. Compared to EGFR, cancer cells rely more on AXL for downstream pro-survival signaling in TNBC [105, 106]. AXL has also been identified as a predictive marker of resistance to EGFR-targeted drugs in triple-negative breast cancer (TNBC) cell lines [105]. In TNBC, AXL is found in close proximity to EGFR, other ErbB receptors and MET, and it can mediate downstream activation of proteins that are crucial for cell migration through a ligand-independent mode of transactivation by EGFR. This crosstalk between EGFR and AXL leads to the activation of pathways that promote cell migration and contribute to drug resistance. Interestingly, the transactivation-mediated RTK signaling might eventually lead to the resistance to receptor-specific-ligand treatment. The heavy reliance of TNBC on AXL or MET, instead of EGFR, for downstream signaling and physical interaction between these RTKs provides clinical rationale for developing therapies targeting AXL or MET signaling.

Distinct signaling through surface receptors and RTK signaling crosstalk after EGFR TKI treatment plays an important role in leading RTK-mediated secondary resistance and provides distinct signaling through new receptors. Future research needs to focus on understanding the mechanisms of receptor family transactivation and how different RTKs collaborate in promoting resistance to come up with alternative strategies to overcome therapeutic resistance.

## MECHANISMS OF ANTI-EGFR RESISTANCE MEDIATED BY RTKS IN HEAD AND NECK CANCER

### Transactivation of EGFR mediated by RON/HGFL (hepatocyte growth factor-like protein)

In head and neck cancer, a major concern regarding targeted therapy is resistance to anti-EGFR based therapies despite high levels of EGFR expression. Cetuximab is FDA approved for treatment of HNSCC, but its efficacy as a monotherapy is limited, and head and neck cancer cells tend to develop resistance over time [43]. A common mechanism of resistance entails cells resorting to RTKs for survival. Overexpression of HGF/MET is commonly observed in resistant head and neck cancer cells and this signaling helps the cells survive despite the detrimental effects of cetuximab. Although MET activation by HGF is primarily driven by paracrine signaling from cancer-associated fibroblasts, it has been found that in tumors resistant to gefitinib and erlotinib, MET can also be activated independent of ligand binding [35, 43].

**Figure 4:**
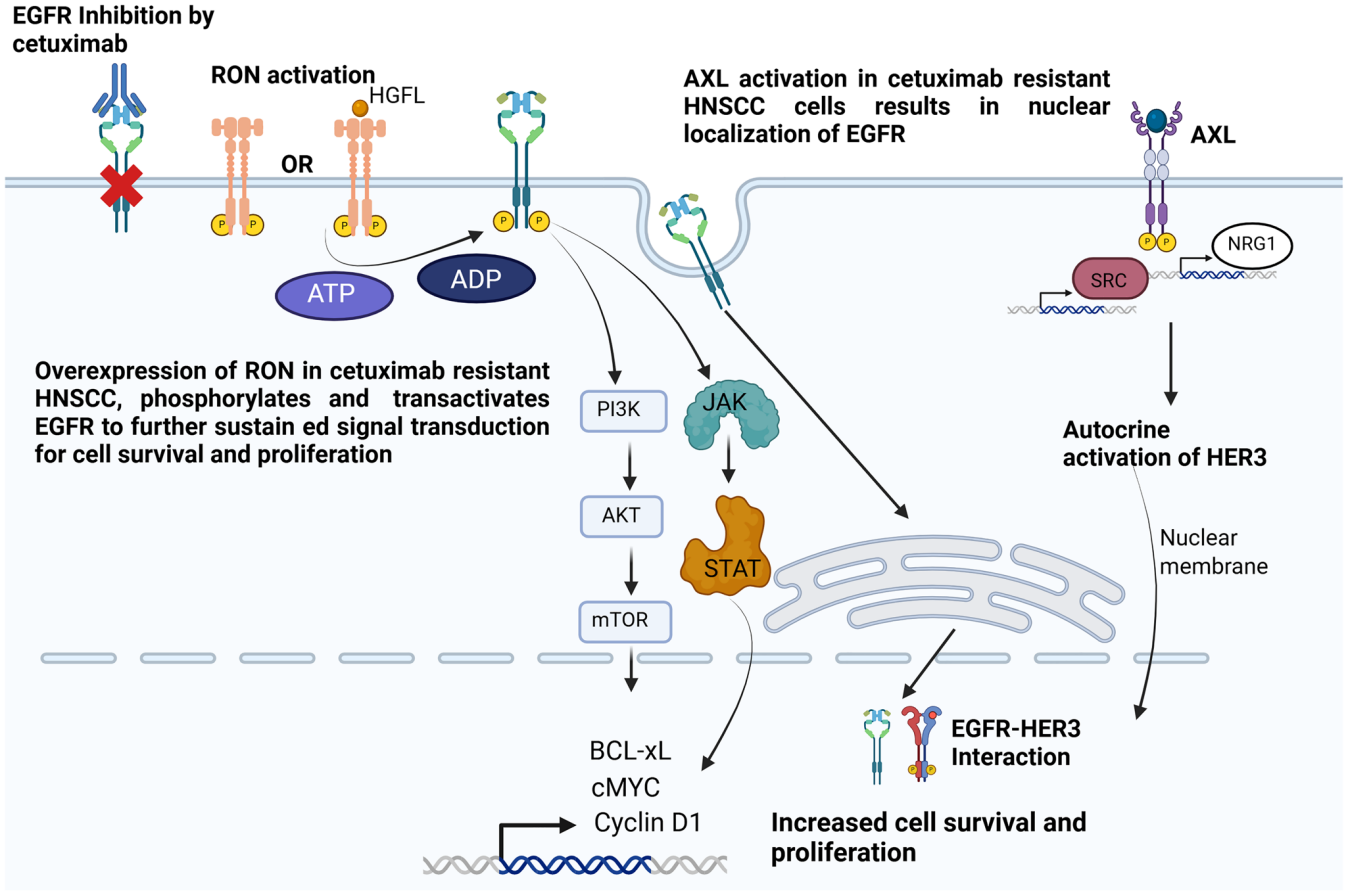
Mechanisms of anti-EGFR therapy resistance mediated by RON/HGFL and AXL RTKs in head and neck cancer. Cetuximab resistant cells tend to overexpress RON RTK which either by homodimerization or binding to extracellular ligands can phosphorylate neighboring EGFR (not bound by cetuximab) and can lead to sustained signal transduction by activating PI3K/Akt/mTOR pathway or JAK/STAT pathway leading to transcription of genes like c-MYC, BCL-XL and Cyclin D1 which prevent apoptosis and increase cell proliferation. Cetuximab resistant cells also show activation of AXL, which promotes EGFR internalization. AXL activation also leads to expression of Src kinases and NRG1 which activates HER3 in an autocrine manner and causes EGFR-HER3 interaction. Created with https://www.biorender.com/.

There is increasing evidence that the RON/HGFL interaction plays an important role in tumorigenesis of various cancers including breast cancer, prostate cancer, and head and neck cancers. RON can be activated by dimerization and phosphorylation and RON RTK signaling can also be independent of the presence of HGFL [107]. Studies have shown that squamous papillomas with defective RON signaling show reduced tumor growth. In HNSCCs, RON expression is associated with active phosphorylation of EGFR. Patients with HNSCC showing expression of RON along with phosphorylated EGFR (pEGFR) had worse event-free survival compared to those without pEGFR and RON [30]. Studies with certain squamous cell carcinoma cell lines also suggest that RON plays an important role in conferring a migratory phenotype in these cells and the downregulation of RON reduces cell migration even when the cells contain functional EGFR. In fact, RON was found to play a synergistic role with EGFR to confer invasive phenotypes in HNSCC. There is also an interesting crosstalk observed between EGFR and RON wherein EGFR gets trans-phosphorylated upon RON activation and there is interaction between activated RON and EGFR (Figure 4) [30, 107].

### AXL mediated signal transduction

Cetuximab resistant HNSCC cells also demonstrate an upregulation and hyperactivation of another type of RTK named AXL. EGFR undergoes nuclear translocation upon activation of AXL and AXL knockdown facilitates inhibition of tumor proliferation by modulating EGFR activity. AXL also promotes expression of Src kinases and NRG1 (Figure 4), which further activates EGFR and its interaction with HER3. Cell lines sensitive to cetuximab became cetuximab resistant upon AXL overexpression. AXL has also been shown to induce epithelial to mesenchymal transition [106].

## MECHANISMS OF ANTI-EGFR THERAPY RESISTANCE MEDIATED BY OTHER FACTORS IN BREAST CANCER

### EGF-related ligand-dependent activation

Unlike specific EGFR mutations found in lung cancer that determine tumor sensitivity/resistance to the EGFR tyrosine kinase inhibitor gefitinib, such mutations are uncommon in breast cancer cells [108, 109]. In breast cancer, ligand-dependent activation of EGFR can contribute to resistance. A study investigated EGF-related ligands of the EGFR family using both gefitinib-sensitive and resistant breast cancer cell lines with varying EGFR expression levels [110]. In gefitinib-resistant cells, the expression and location of EGF-related ligands change, with the ligands translocating into the nucleus and interacting with genes involved in transcriptional regulation upon EGFR inhibition. This nucleo-cytoplasmic trafficking of EGFR ligands may counterbalance the loss of EGFR function and play a crucial role in determining sensitivity to EGFR TKI treatment. Additionally, fibroblast secretion of HGF was found to activate MET and lead to EGFR/MET crosstalk, resulting in resistance to EGFR TKIs in triple-negative breast cancer [111]. Tumor-stromal interactions appear to contribute to the intrinsic sensitivity of breast cancer cells to EGFR TKIs, providing an alternative approach to confer EGFR therapy resistance in breast cancer.

### Nuclear EGFR

**Figure 5:**
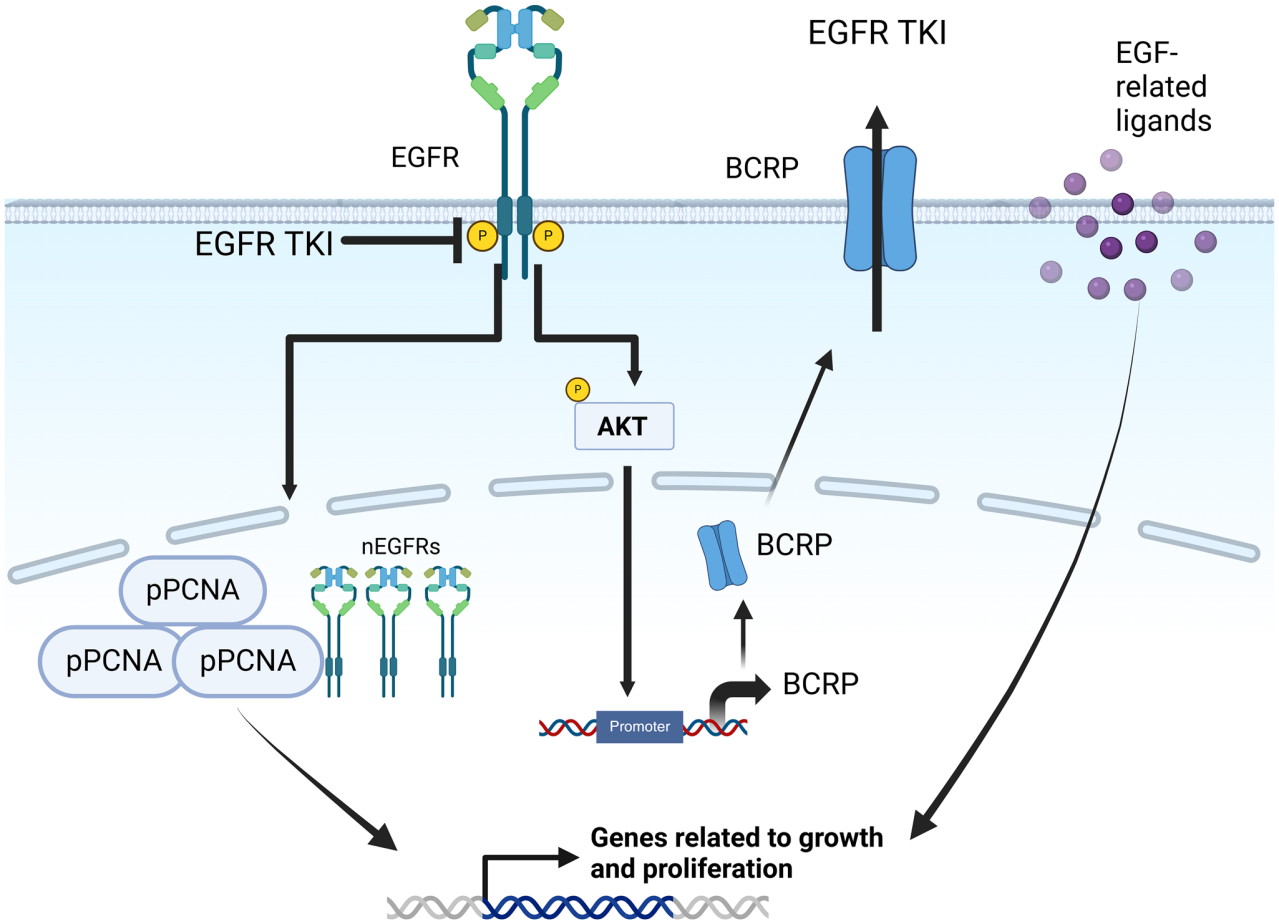
Mechanisms of anti-EGFR therapy resistance in breast cancer via EGF-related ligands and nuclear EGFR. Upon EGFR TKI treatment, EGFR can directly translocate into the nucleus and stabilizes PCNA in order to promote cell growth and proliferation. EGFR can also activate Akt and transcribe the breast cancer-resistant protein (BCRP), an ATP-binding cassette efflux transporter to eliminate EGFR TKIs from the cancer cells. Created with https://www.biorender.com/.

In wild-type EGFR-expressing cancer cells, mechanisms of the resistance to gefitinib remain largely unknown. Studies have shown the nuclear localization of EGFR, where it functions as a transcription factor required for DNA repair and resistance to cisplatin treatment [112, 113]. As shown in Figure 5, upon EGFR-TKI treatment, the level of nuclear EGFR in wild-type EGFR-expressing breast cancer cells increases [112]. This nuclear translocation is mediated by Akt phosphorylation of EGFR (Figure 5). Nuclear EGFR enhances the transcription of the breast cancer-resistant protein (BCRP) gene by recruiting to its promoter region, potentially contributing to EGFR TKI therapy resistance. Nuclear EGFR has also been found to phosphorylate and stabilize proliferation cell nuclear antigen (PCNA). Increased PCNA expression has been linked to a poor prognosis in breast cancer patients, and EGFR TKI-resistant breast cancer cells show elevated PCNA expression [113]. Treatment with cell-penetrating PCNA peptide induced apoptosis in the cells and prevented nuclear EGFR binding to PCNA *in vivo* [114, 115]. There is an urgent need to study the functional impact of nuclear EGFR in breast cancer growth, migration, and therapeutic response to EGFR-targeted therapies.

## MECHANISMS OF ANTI-EGFR THERAPY RESISTANCE MEDIATED BY OTHER FACTORS IN HEAD AND NECK CANCER

### Activation of TLR4

Resistance to anti-EGFR therapies in head and neck cancer not only involves overexpression and activation of RTKs but also other factors, such as loss of tumor-suppressor genes, signaling by pattern recognition receptors, and activation of inflammatory pathways. Inflammatory cytokines such as IL8 and IL1B have been reported to promote resistance to anti-EGFR therapies in other cancers such as colorectal cancer. In HNSCC tissue biopsies expressing EGFR, there are elevated levels of the innate immune receptor, Toll-Like Receptor 4 (TLR4), which is involved in recognizing self-proteins and inflammatory microbes [116]. TLR4 is also upregulated in ovarian, breast, and other hormone responsive tumors.

**Figure 6:**
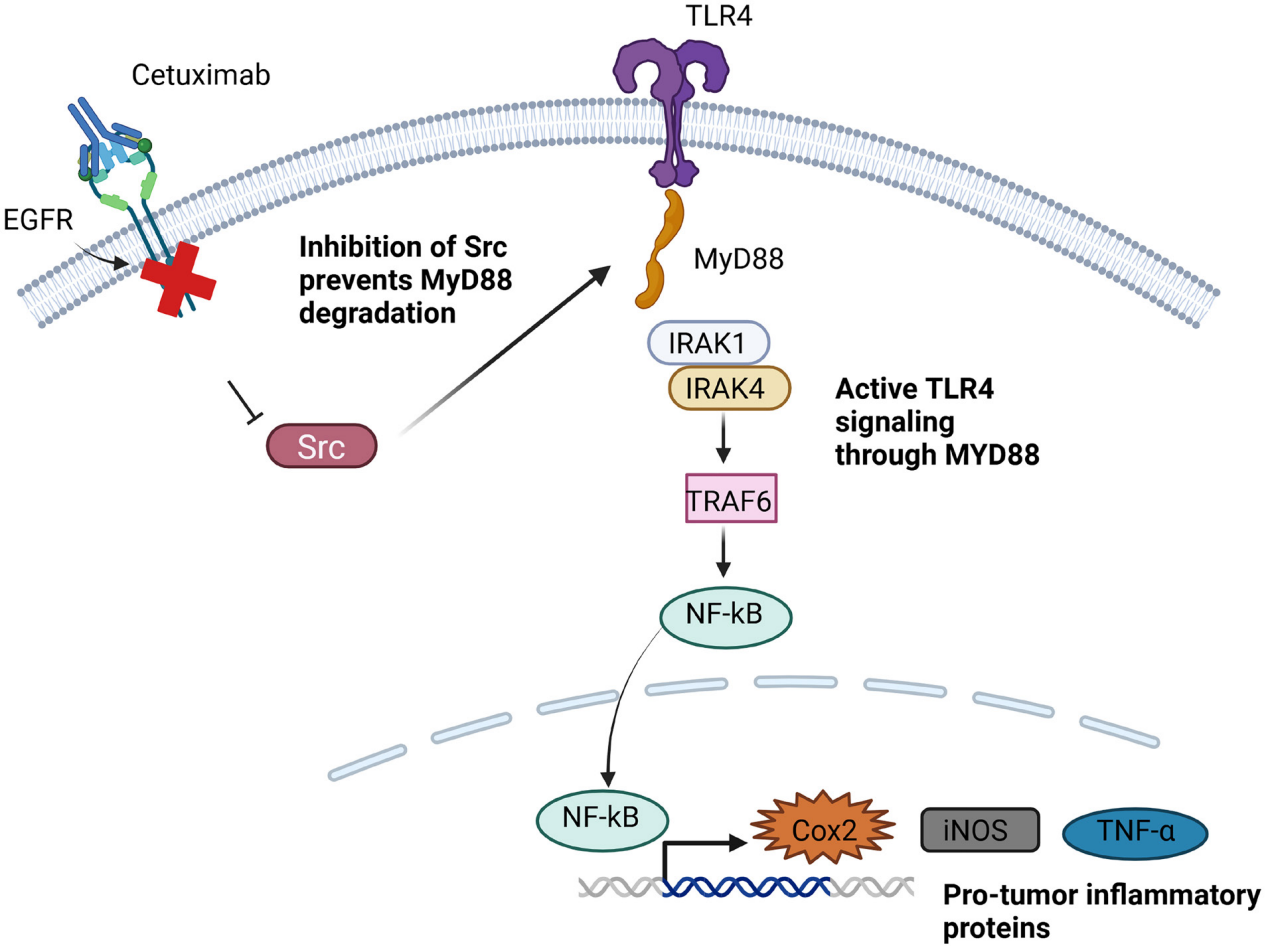
Mechanism of Anti-EGFR resistance in head and neck cancer by activation of TLR4-MyD88 signaling. EGFR inhibition by cetuximab prevents Src activation, thus preventing MyD88 degradation, leading to activation of TLR4 signaling via NFKB, leading to production of anti-apoptotic and pro-tumor inflammatory proteins that promote cancer cell survival. Created with https://www.biorender.com/.

When TLR4 is activated and dimerized it leads to the activation of NF-kB resulting in the upregulation of cell survival proteins. Interestingly, the blockage of EGFR with cetuximab has been shown to activate TLR4 in a MyD88 dependent manner (Figure 6). Overexpressing TLR4 in HNSCC cells makes them more resistant to cetuximab both *in vitro* and *in vivo* [117]. In HNSCC cells overexpressing MyD88, there is an upregulation of pro-tumor inflammatory proteins including iNOS, TNF-α, and COX2. Cetuximab binding to EGFR prevents Src activation, which in turn decreases MyD88 degradation. Consequently, MyD88 activates TLR4 signaling, leading to the expression of anti-apoptotic proteins, and increased pro-tumor inflammation [117]. This provides a route for cancer cells to resist EGFR inhibition and promotes cell survival. Understanding these non-RTK-mediated mechanisms of resistance is crucial for developing effective strategies to overcome resistance to EGFR-inhibiting treatments in head and neck cancer.

### Loss of PTEN

Blocking of EGFR signaling by inhibitors like cetuximab activates the PI3K/Akt/mTOR pathway, which is regulated by PTEN (Phosphatase and tensin homolog), a tumor suppressor gene. Loss of PTEN expression or function occurs in about one-third of HNSCC patients, which allows the cells to resort to the PI3K/Akt/mTOR pathway to survive, thus diminishing cetuximab’s clinical benefits [118]. Thus, patient tumors with endogenous PTEN deficiency are found to be more resistant to cetuximab treatment than those harboring PTEN. This makes PTEN gene copy number loss a predictive biomarker for cetuximab treatment resistance [119]. Experimental evidence suggests that WWP1 which is an E3 ubiquitin ligase is involved in the negative regulation of PTEN expression. Genetic ablation of WWP1 has shown to reverse the downregulation of PTEN and led to subsequent inhibition of PI3K/Akt/mTOR pathway of cell survival. Therefore, therapeutic drugs targeting WWP1 may be able to reverse the cetuximab resistance caused by PTEN loss [120].

### Activation of STAT3 and PI3K signaling pathway

*In-vitro* studies have shown that head and neck cancer cell lines that are resistant to cetuximab have different responses to stimulation by EGF. The UMSCC1 cell line, when made resistant to cetuximab, tends to show enhanced downstream signal transduction upon EGF stimulation, while the UMSCC6 cell line resistant to cetuximab had the potential to activate downstream signaling even in absence of EGF [121]. The knockdown of STAT3 promotes pro-apoptotic pathways in cetuximab resistant UMSCC1 cell lines. In fact, even in the absence of cetuximab treatment, STAT3 knockdown has an anti-proliferative effect in UMSCC1 head and neck cells [121]. Additionally, cetuximab resistant tumors have activating mutations and gene amplifications in the PI3K pathway. Studies on HNSCC patient-derived xenograft models demonstrated that the combinatorial treatment of cetuximab and the PI3Kα and δ inhibitor, Copanlisib, had markedly noticeable therapeutic effect in HNSCC tumors, especially those resistant to cetuximab [122].

## FUTURE DIRECTIONS

Receptor tyrosine kinases play a critical role in regulating cancer cell growth and metastasis. Over the past few decades, there has been increasing interest in developing small molecules that target RTKs as potential therapies for various types of cancer, with a particular focus on EGFR in different cancer types. However, the effectiveness of targeted therapy is often hindered by intrinsic and extrinsic resistance and adverse effects, as well as by adverse effects. For instance, EGFR inhibitors have shown limited efficacy in breast cancer patients, and frequent incidences of therapy resistance have been observed in head and neck cancer with EGFR-targeted inhibitors.

Therefore, a key area of research should be investigation of common resistance mechanisms of EGFR inhibitors, where RTKs like IGF-1R, MET, and AXL compensate for EGFR inhibition and promote survival signaling cascades. Investigating combinatorial approaches that involve inhibiting both EGFR and other components of oncogenic pathways, such as MET, RON, and AXL, holds promise in overcoming resistance to anti-EGFR therapeutics. A recent phase 1 study has shown that patients with recurrent or metastatic head and neck cancer who received BCA101, a bifunctional dual targeting drug that targets EGFR and TGF-β in combination with pembrolizumab, were able to achieve an overall response rate of 65%. Thus, there seems to be an interesting crosstalk of EGFR-TGF-β and PD-1 which could be further explored in head and neck cancers and other cancer types treated with anti-EGFR or pembrolizumab alone [123]. Additionally, less common mechanisms regulated by non-RTKs, such as loss of tumor suppressor genes, activation of inflammatory pathways, and alterations in downstream signaling pathways, need to be explored further to enhance our knowledge of therapy sensitivity in breast cancer, head and neck cancer and other cancers where EGFR plays a pivotal role in promoting tumorigenesis. Understanding the interplay between different signaling pathways and their impact on cancer progression and treatment is essential in designing more effective combination therapies. Moreover, the development of reliable biomarkers that can predict resistance to EGFR therapy is essential. These biomarkers will help clinicians identify patients who are likely to be resistant to EGFR therapy, enabling them to choose alternative treatment approaches or effective combinatorial treatments and improve patient outcomes. By targeting multiple pathways simultaneously, we can potentially overcome resistance and improve the overall efficacy of EGFR-targeted therapies in cancer treatment.

## COMBINING EGFR-TARGETED THERAPY WITH IMMUNE CHECKPOINT BLOCKADE: CURRENT CLINICAL TRIALS AND FUTURE THERAPEUTIC POTENTIAL

### Breast cancer

Multiple clinical trials have explored immune checkpoint inhibitors (ICIs) in breast cancer, particularly in TNBC patients [124–134]. Currently, two FDA-approved combination therapies: pembrolizumab (a PD-1 antibody) and atezolizumab (a PD-L1 antibody), are used alongside chemotherapy for selected breast cancer patients. These combination strategies have demonstrated significant improvements in progression-free survival compared to placebo-chemotherapy, with acceptable safety profiles [135, 136]. In inflammatory breast cancer (IBC), an immunosuppressive tumor microenvironment mediated by EGFR signaling may contribute to ICI resistance [137]. Notably, the combination of panitumumab (an EGFR monoclonal antibody) with an ICI has a more substantial inhibitory effect on tumor growth than ICI alone. Furthermore, the efficacy of EGFR-targeted therapy appears to be associated with T-cell-mediated immune responses, suggesting that combining EGFR inhibitors or antibodies with ICIs could offer a promising therapeutic approach for BC patients with high EGFR copy numbers.

### Head and neck cancer

EGFR inhibition in HNSCC by cetuximab is known to promote antibody-dependent cellular cytotoxicity by recruiting natural killer (NK) cells and also elevates the numbers of certain T cell populations namely PD-1 and TIM-3 positive CD8+ T cells [138]. Thus, the addition of ICIs with EGFR inhibition leverages both direct tumor cell cytotoxicity and the activation of anti-tumor immunity, potentially aiding in overcoming resistance to EGFR inhibition alone. Multiple clinical trials have investigated combining cetuximab with ICIs, particularly in Recurrent/metastatic (R/M) HNSCC patients (Table 3) [139–143].

**Table 3:** Key clinical trials evaluating cetuximab and immune checkpoint inhibitors in HNSCC

Treatment	Immune checkpoint target	Trial	Cancer subtype	Trial phase	Primary endpoint	Outcome
Durvalumab+ Cetuximab	PD-L1	NCT03691714 [139]	Recurrent/metastatic (R/M) HNSCC	II	Objective Response Rate (ORR)	ORR = 39%
Pembrolizuma+ Cetuximab	PD-1	NCT03082534 [140]	Recurrent/metastatic (R/M) HNSCC	II	Overall Response rate (ORR)	ORR = 45%
Nivolumab+ Cetuximab	PD-1	NCT03370276 [141]	Recurrent/metastatic (R/M) HNSCC	II	Overall Response rate (ORR)	Median overall survival (patients with prior therapy for R/M HNSCC): 11.4 months
						Median overall survival (patients with no prior therapy): 20.2 months
Cetuximab+ Camrelizumab	PD-1	NCT05673577 [142]	Recurrent/metastatic (R/M) HNSCC	II	Overall response rate (ORR)	ORR = 90.5%
Monalizumab+ Cetuximab	NKG2A	NCT02643550 [143]	Recurrent/metastatic (R/M) HNSCC	I	Overall response rate (ORR)	ORR = 20%

## Abbreviations

AREG: Amphiregulin
AXL: receptor tyrosine kinase
BRCP: Breast cancer-resistant protein
EGFR: Epidermal Growth Factor Receptor
FAK: Focal Adhesion Kinase
FGFR: Fibroblast Growth Factor Receptor
HGF: Hepatocyte Growth Factor
HGFL: Hepatocyte Growth Factor-Like protein (also known as MSP - Macrophage Stimulating Protein)
HER: Human Epidermal Growth Factor Receptor (commonly refers to HER2)
HNSCC: Head and Neck Squamous Cell Carcinoma
IGF: Insulin-like Growth Factor
iNOS: Inducible Nitric Oxide Synthase
JAK: Janus Kinase
MAPK: Mitogen-Activated Protein Kinase
MET: Proto-Oncogene, Receptor Tyrosine Kinase
MSTR1: Macrophage Stimulating 1 Receptor (same as RON)
NF-kB: Nuclear Factor kappa-light-chain-enhancer of activated B cells
PCNA: Proliferating Cell Nuclear Antigen
PDGFR: Platelet-Derived Growth Factor Receptor
PTEN: Phosphatase and Tensin Homolog
RON: Recepteur d’Origine Nantais (same as MST1R)
RTK: Receptor Tyrosine Kinase
TGF-α: Transforming Growth Factor-alpha
TLR: Toll-Like Receptor
TNBC: Triple-Negative Breast Cancer
TNF-α: Tumor Necrosis Factor-alpha
VEGF: Vascular Endothelial Growth Factor
